# Intra-Specific Difference in the Effect of Salinity on Physiological Performance in European Perch (*Perca fluviatilis*) and Its Ecological Importance for Fish in Estuaries

**DOI:** 10.3390/biology8040089

**Published:** 2019-11-17

**Authors:** Emil A. F. Christensen, John D. Stieglitz, Martin Grosell, John F. Steffensen

**Affiliations:** 1 Marine Biological Section, Department of Biology, University of Copenhagen, Strandpromenaden 5, 3000 Elsinore, Denmark; jfsteffensen@bio.ku.dk; 2 Department of Marine Ecosystems and Society, Rosenstiel School of Marine and Atmospheric Science, University of Miami, 4600 Rickenbacker Causeway, Miami, FL 33149, USA; jstieglitz@rsmas.miami.edu; 3 Department of Marine Biology and Ecology, Rosenstiel School of Marine and Atmospheric Science, University of Miami, 4600 Rickenbacker Causeway, Miami, FL 33149, USA; mgrosell@rsmas.miami.edu

**Keywords:** cost of osmoregulation, critical swimming speed, maximum metabolic rate, oxygen consumption rate, standard metabolic rate, static respirometry, swimming respirometry

## Abstract

Changes in environmental salinity challenge fish homeostasis and may affect physiological performance, such as swimming capacity and metabolism, which are important for foraging, migration, and escaping predators in the wild. The effects of salinity stress on physiological performance are largely species specific, but may also depend on intra-specific differences in physiological capabilities of sub-populations. We measured critical swimming speed (U_crit_) and metabolic rates during swimming and at rest at salinities of 0 and 10 in European perch (*Perca fluviatilis*) from a low salinity tolerance population (LSTP) and a high salinity tolerance population (HSTP). U_crit_ of LSTP was significantly reduced at a salinity of 10 yet was unaffected by salinity change in HSTP. We did not detect a significant cost of osmoregulation, which should theoretically be apparent from the metabolic rates during swimming and at rest at a salinity of 0 compared to at a salinity of 10 (iso-osmotic). Maximum metabolic rates were also not affected by salinity, indicating a modest tradeoff between respiration and osmoregulation (osmo-respiratory compromise). Intra-specific differences in effects of salinity on physiological performance are important for fish species to maintain ecological compatibility in estuarine environments, yet render these sub-populations vulnerable to fisheries. The findings of the present study are therefore valuable knowledge in conservation and management of estuarine fish populations.

## 1. Introduction

Most aquatic species only tolerate and perform well in the stable environmental salinities of either fresh water or sea water [1,2]. Consequently, species diversity is highest in either fresh or marine habitats, and decreases from both ends of the salinity gradient towards intermediate salinities in estuaries, which constitute a mixing zone between fresh water and oceanic seawater [3]. Estuaries usually have high biological productivity due to nutrient input from terrestrial run-off [4] and the high food availability and low inter-specific competition may be a strong driving force for physiological adaptation to tolerate and perform well at intermediate salinities [5,6]. Adaptation to diverging environments develops on intra-specific level either through adaptive phenotypic plasticity, that is, the ability of one genotype to produce multiple phenotypes in response to different ambient environments [7], or through genetic adaptation [8,9]. Such intra-specific differences in physiological performance can be crucial for species for maintaining high fitness in novel environments [8,10].

The European perch (*Perca fluviatilis*) is a freshwater fish native to the major part of Eurasia and, furthermore, has been introduced in parts of southern Europe, Africa, Australia, and New Zealand [11,12,13]. Although considered a stenohaline species, it also inhabits brackish waters in estuaries such as the Baltic Sea, one of the world’s largest estuaries. European perch is an ecologically important predator with a significant top-down regulating effect on the environment it inhabits [14,15]. However, its predatory lifestyle is a major issue in Australia, where it preys on endemic species and therefore is considered invasive [16]. In Europe, the species is popular for human consumption, and consequently a target for both a significant commercial and recreational fishery, as well as development in aquaculture rearing [12,13,17]. However, the abundance of European perch in the Baltic Sea has declined markedly in recent decades, probably owing to anthropogenic pollution of the environment [15,18,19]. A recent study showed that European perch originating from brackish water in the Baltic Sea had a markedly higher maximum salinity tolerance and an enhanced osmoregulatory capability compared to European perch originating from an inland freshwater lake, which has been supported by genetic divergence between the populations in these two habitats [17,20]. However, it remains unknown whether this intra-specific difference in salinity tolerance and osmoregulatory capability translates into an alternated physiological performance that could be an ecological advantage for the species in estuaries. European perch thus constitutes an ideal candidate for studying intra-specific difference in the effect of salinity change on physiological performance and to evaluate its ecological importance.

In teleost fish, osmotic water movement and ion diffusion can occur over gills and skin, where permeability is required for respiratory gas exchange [21]. Fish regulate their internal osmotic pressure to around 3–350 mOsm kg^−1^ to maintain homeostasis regardless of the ambient salinity, and even minor osmotic imbalances can impair physiological performance or be lethal [21,22]. Changes in internal osmotic pressure in response to environmental salinity can affect the physiological performance of fish, such as swimming performance [23,24], which is an important trait for fish to migrate, forage, and escape predators in the wild [25,26]. Although osmotic distress is presumably lowest where the ion and water gradients between the ambient water and the internal milieu approach zero, only some studies have shown critical swimming speed (U_crit_; the maximum prolonged swimming speed) to be highest in iso-osmotic water conditions, that is, at a salinity of around 10 [23,24,27], while other studies have shown the highest U_crit_ in either fresh or sea water or no effect of salinity on U_crit_ at all [5,28,29,30,31,32]. Salinity change may also induce an energetic load through an added cost of osmoregulation. This should be apparent from the standard metabolic rate (SMR), that is, the temperature specific metabolic rate of a resting, non-digesting fish [33], which should in theory be lowest in iso-osmotic conditions and increase at salinities departing from this level [31,34,35,36]. Furthermore, the cost of osmoregulation may also depend on the activity level of the fish. Activity increases ventilatory flow to meet elevated oxygen demands, which enhances the potential for diffusion of water and ions over the gills, and, in turn, increases the energetic need for osmoregulation [34,35,37]. However, as for U_crit_, there is no consensus around near iso-osmotic conditions being favorable for the metabolism of fish, as studies have also shown that metabolic rates are not necessarily lowest at this level [38]. Neither is there agreement on the magnitude of the cost of osmoregulation, which varies from not measurable on SMR, to more than 30% of SMR. In addition, fish may also reduce the permeability of their gills to limit osmotic water movement and ion diffusion, at the cost of reducing respiratory gas exchange, a phenomenon known as the osmo-respiratory compromise [39]. This may have energetic implications by reducing the maximum metabolic rate (MMR), that is, the highest achievable metabolic rate of a fish at a given temperature [40]. Both increased SMR and reduced MMR may reduce the aerobic scope, that is the magnitude by which an animal can increase its metabolic rate above that of SMR, in turn limiting the capacity for performing crucial activities in fish, such as swimming and digestion [41].

The responses of fish swimming performance and metabolic rates to salinity changes are largely species specific, but also depend on the ability of sub-populations to cope with changing salinity [5,28,29]. Such intra-specific difference has been shown in a variety of species, and is considered important for obtaining ecological compatibility, colonizing, and diversifying in habitats of different salinities [6,20,28,29,42,43]. In the present study, we measured U_crit_ and whole-animal metabolism by means of oxygen consumption rates (ṀO_2_), while swimming and at rest, at salinities of 0 and 10 in European perch. The fish originated from a low salinity tolerant population (LSTP) from a freshwater lake and from a high salinity tolerant population (HSTP) from a brackish water estuary [20]. It was hypothesized that iso-osmotic conditions (a salinity of 10) would be favorable for swimming performance due to lower osmotic gradient between the environment and internal milieu of the fish, and thus minimal osmotic distress. Furthermore, ṀO_2_ was expected to be highest in fresh water due to the cost of osmoregulation and that the cost of osmoregulation would increase with increasing swimming speed due to a higher potential for osmotic diffusion caused by increase in ventilation rate. In addition, we anticipated that HSTP would perform best along the salinity gradient, as the population has superior capabilities to cope with salinity changes.

## 2. Materials and Methods

### 2.1. Animals and Holding

Permission to catch wild European perch was given by the Danish AgriFish Agency (reference number: 12-7410-000008), and the experimental protocols were approved by The National Authority (journal number: 2012-15-2934-00657). Fish holding and experiments were conducted at the Marine Biological Section, University of Copenhagen, Elsinore, Denmark. Fish from LSTP (*N* = 8, body masses (BM) = 104 ± 7 g (average ± SE) and total lengths (TL) = 21.0 ± 0.4 cm) were caught by angling in the freshwater Lake Esrom (55°58′09′′ N; 12°22′08′′ E). Fish from HSTP (*N* = 8, BM = 109 ± 7 g, and TL = 20.8 ± 0.4 cm) were caught by angling in the western Baltic Sea (55°27′19′′ N; 12°11′56′′ E). The salinity in this area is on average 10 [44,45,46], but fluctuates considerably between 0 to 22 over time due to tides, stratification, patterns in river run-off, and periods of heavy winds, either forcing the high salinity water from Kattegat into the Baltic Sea or low salinity water from the Baltic Sea into Kattegat [47,48,49]. The fish were tagged with passive integrated transponders in the dorsal musculature to allow for identification, and were kept in 160 L aquaria, with four fish in each, at a water temperature of 20 °C. Before any acclimation or experimentation commenced, the fish were kept in fresh water (unchlorinated Elsinore tap water with a salinity of 0.4, henceforth rounded off to a salinity of 0) for three weeks to acclimate to the laboratory facilities and start eating. The fish were fed sliced herring twice a week. To maintain proper water quality, excess food was siphoned out, 60% of the water was renewed every week, and the water thoroughly aerated and continuously filtered through a trickle filter. To minimize stress, artificial seaweed and pipes were used as shelter structures and the light level kept low. The lighting period was 12 h light, 12 h dark.

### 2.2. Experiments

Two experimental trials were conducted at salinities of 0 (fresh water) and 10 (iso-osmotic water): a U_crit_/swimming respirometry trial and a static respirometry trial. The experiments were conducted repeatedly on the same individuals at the two salinities. This approach was chosen to minimize inter-individual variation as the effects of salinity on physiological performance and metabolism may be subtle in fish [38,50]. The fish were acutely transferred to the desired salinity, and subsequently acclimated for one month before being experimented on. After having gone through both experimental trials at the first salinity treatment, the fish were acutely transferred to the other salinity treatment and acclimated for another month before being experimented on again. To allow testing for carryover effects of the repeated measurement half of these individuals experienced the salinities in the order 0–10, the other half in the order 10–0. Brackish water was obtained by mixing tap water with filtered sea water from Oresund with a salinity of around 30. The salinity was checked daily with a handheld meter (WTW Multi 3410 conductivity meter, Xylem Analytics, Weilheim, Germany) and adjusted as necessary. The fish were fasted 5 days prior to experimentation, and their BM, TL, depth (D), and width (W) were measured before each experiment.

All experiments were conducted on individual fish. At each salinity, the fish was first placed in a 11.8 L Steffensen Mk III swimming respirometer, set up as in [31], at a swimming speed of 0.5 body length per second (BL s^−1^). The fish was left undisturbed overnight (between 15 and 20 h) to recover from handling and to acclimate to the respirometer. During the next day, the fish was subjected to a U_crit_ swimming protocol [51] where the speed was increased with increments of 0.5 BL s^−1^ every 40 min until fatigue (U_crit_). Fatigue was determined as the point where the fish had rested against the rear grid of the swimming section in the respirometer for more than 10 s. ṀO_2_ was measured at each speed with a fiber optic oxygen sensor (Fibox 3, Presence Precision Sensing GmbH, Regensburg, Germany) by automated intermittent-flow respirometry, which allows for multiple and precise measurements of ṀO_2_ over long time series [52,53]. Two ṀO_2_ measurements were taken for each speed. The flush time was 330 s to ensure a 95% water exchange between measurements, the wait time was 150 s to ensure proper mixing of the water in the respirometer before measurements, and the measuring time was 720 s to ensure a regression R^2^ of >0.95 of the linear decrease in oxygen partial pressure per time. One background respiration (BR) measurement was taken after each trial at a water speed of 0.5 BL s^−1^, without the presence of the fish. The BR measurement was taken immediately after removing the fish with a wait period of 300 s and a measuring time of 2400 s. The long measurement time for BR measurement was necessary to ensure a regression R^2^ of above 0.95 due to low decrease in oxygen partial pressure per time [53].

After the U_crit_ and respirometry trial, the fish was directly transferred to a custom-made, cylindrical, acrylic static respirometer of 1.430 L, set up as in [54]. Here, ṀO_2_ was measured over a period of 22–24 h with a fiber optic oxygen sensor (Firesting Pro 1 channel, PyroScience GmbH, Aachen, Germany) by automated intermittent-flow respirometry [53], allowing for estimation of SMR [33]. The flush time was 300 s, the wait time 60 s, and the measuring time 150 s. Three hours into the static respirometry trial, the fish was manually stressed by turning on the light in the otherwise dimly lit experimental room and lightly tapping on the respirometer for 3 min to elicit an estimate of MMR. Background respiration (BR) was measured after each trial without the presence of the fish with a wait period of 200 s and a measuring time of 1800 s to ensure a regression R^2^ of above 0.95.

### 2.3. Data Analysis and Statistics

U_crit_ was calculated as:

U_crit_ = U_i_ + t_i_ × t_ii_^−1^ × U_ii_ (1)

where U_i_ is the last swimming speed the fish completed, t_i_ is the time the fish endured at the last swimming speed, t_ii_ is the time interval for each swimming speed, and U_ii_ is the speed increment [51]. ṀO_2_ was calculated for each measurement period by linear regression of oxygen partial pressure (pO_2_) over time (α):

ṀO_2_ = α × V_r_ × β × BM^−1^ (2)

where V_r_ is the respirometer volume (total volume (V_t_) minus animal volume, where animal BM is assumed equal to animal volume), and β is the oxygen solubility constant at the given salinity and temperature [53]. ṀO_2_ measurements were corrected for BR (ṀO_2,corr_) according to [53]:

ṀO_2,corr_ = β × (α_a_ × V_r_ − α_b_ × V_t_) × BM^−1^ (3)

where α_a_ is the linear decline in pO_2_ of a measurement with the animal present, and α_b_ is the linear decline in pO_2_ of BR. Measurements with regression R^2^ lower than 0.95 were excluded from the data analyses. For each individual swimming trial, ṀO_2_ as a function of swimming speed was fitted to a two-parameter exponential function:

ṀO_2_(U) = SMR × e^k × U^ (4)

where k is a constant and U is swimming speed [51]. The function was used to extrapolate SMR and MMR, which was defined as the ṀO_2_ at a swimming speed of 0 BL s^−1^ and ṀO_2_ at U_crit_, respectively [51]. The aerobic scope during swimming was expressed as the difference between these MMR and SMR determinations. The cost of transport (COT, µgO_2_ kg^−1^ BL^−1^) for each swimming speed was calculated as:

COT(U) = ṀO_2_ × U^−1^ (5)

The swimming speed with the lowest cost of transport, that is, the optimal swimming speed (U_opt_), was calculated from Equation (4) according to [55]:

U_opt_ = k^−1^ (6)

The ṀO_2_ measurements from the individual static respirometer trials were sorted into a frequency distribution and a double Gaussian distribution fitted numerically to the histogram [33,56]. SMR from the static respirometry trial was determined as the lowest mean of the two distributions. The other distribution represented spontaneous activity, which was thus excluded from the SMR determination. MMR from the static respirometry trial was defined as the highest measured ṀO_2_ [54]. Aerobic scope from the static respirometry was calculated at the difference between MMR and SMR during the trial.

All statistics were computed in R [57], and the significance level set to an α value of 0.05. Variance homogeneity was confirmed with Bartlett’s test, and data normality tested with the Shapiro–Wilk test. In some instances, data needed log-transformation to obtain normal distribution.

Linear mixed models (LMM) were used to test BM and TL in relation to population, salinity treatment, time (first salinity treatment vs. second salinity treatment, one month apart), and salinity treatment order (0 to 10 vs. 10 to 0), taking into account repeated measurements on the individuals. U_crit_ was analyzed by means of general linear models (GLMs) with repeated measurements on the individuals and in relation to time to test for repeatability and the salinity treatment order to test for carryover effects. SMR, MMR, and aerobic scope were analyzed with a linear mixed model in relation to respirometry methods (swimming and static), salinity, time, and salinity treatment order, taking into account repeated measurement on the individuals. ṀO_2_ during swimming and COT were also analyzed with LMMs with repeated measurements on the individuals, including swimming speed, salinity, time, salinity treatment order and the interaction between swimming speed and salinity to test if ṀO_2_ increased unproportionate with swimming speed due to the added cost of osmoregulation. To exclude effects of potential intra-specific differences in morphometrics on swimming performance and the associated energetics [58], which were not quantified in the present study, the physiological responses were analyzed separately for each population, and any effects only compared qualitatively between the populations.

We conducted a power analysis to estimate the level of cost of osmoregulation on SMR the present study could statistically detect and the sample size needed to enable detection of a cost of osmoregulation of 5% SMR [59,60,61]. The analyses were conducted in Sample Power 3 (IBM, Armonk, NY, USA) using a cut-off power value of 0.8 [62].

## 3. Results

**Figure 1:**
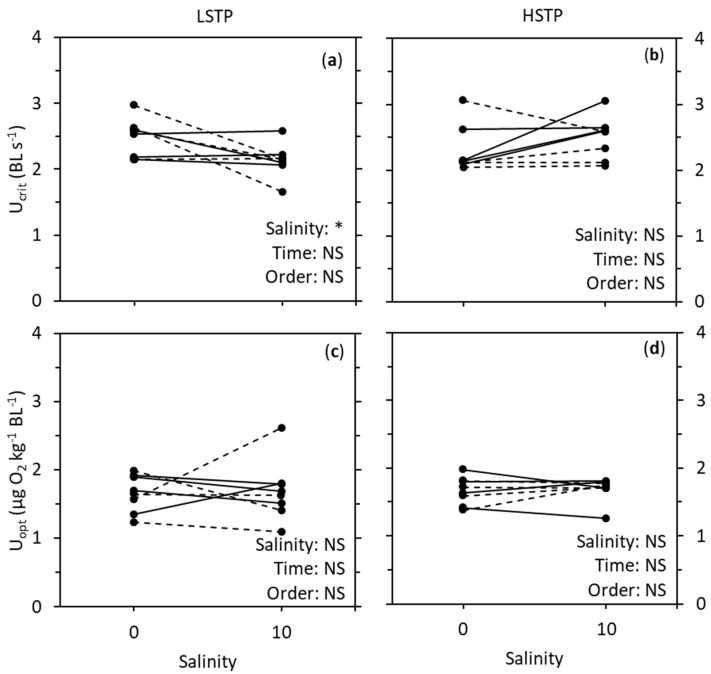
Critical swimming speed (U_crit_; (**a**,**b**)) and optimal swimming speed (U_opt_; (**c**,**d**)) of European perch (*Perca fluviatilis*) from a low salinity tolerance population (LSTP; (**a**,**c**)) and a high salinity tolerance population (HSTP; (**b**,**d**)). “BL” is body lengths. U_crit_ and U_opt_ was determined at salinities of 0 and 10. The total sample size was 16, with 8 individuals being used repeatedly at both salinities. Half the individuals experienced the salinity treatments in the order 0–10 (full lines), the other half in the order 10–0 (dashed lines). The results were analyzed with a general linear model which included salinity, time (first salinity treatment vs. second salinity treatment, one month apart), and the order of which the experiments were repeated on the fish. “*” and “NS” represent *p* < 0.05 and not significant, respectively.

BM and TL size were not significantly different between populations, salinities, time, or salinity treatment order. U_crit_ was significantly affected by salinity in LSTP (GLM, *F*_1,6_ = 7.120, *p* = 0.037), being 16% lower at a salinity of 10 compared to in fresh water (Figure 1, Table 1). Although salinity change seemed to affect U_crit_ more in the group of fish that started at a salinity of 10, there were no effects of either time nor salinity treatment order. However, a decrease in U_crit_ with increasing salinity was not apparent in HSTP, where U_crit_ was unaffected by salinity, time, and salinity treatment order. In both populations, U_opt_ were neither affected by salinity, time, nor salinity treatment order.

**Figure 2:**
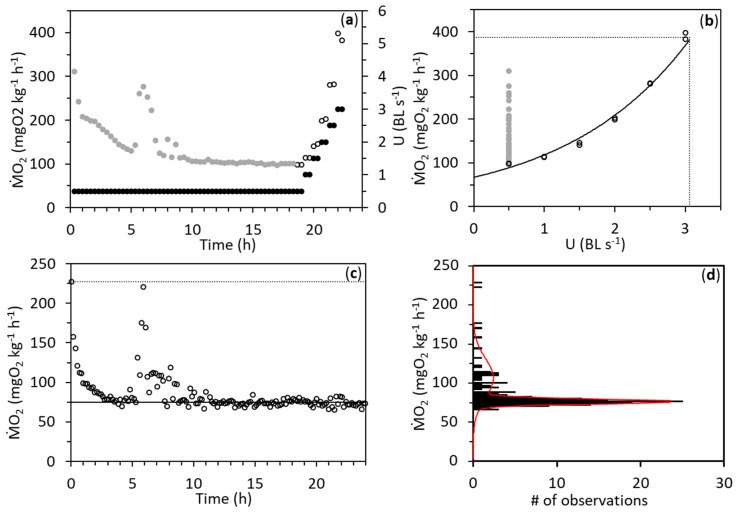
Oxygen consumption rates (ṀO_2_) of a European perch (*Perca fluviatilis*) of 125 g and 22 cm from a high salinity tolerant population at a salinity of 10. (**a**) ṀO_2_ over time in a swimming respirometry trial, where swimming speed (U) is given in body lengths (BL) s^−1^. Grey circles represent ṀO_2_ before a critical swimming speed test, where ṀO_2_ is represented by open circles. (**b**) ṀO_2_ from the swimming respirometry trial in relation to U. A two-parameter exponential function was fitted to ṀO_2_ vs. U during the U_crit_ test (solid line). Standard metabolic rate (SMR) was estimated by extrapolating this fit to a swimming speed of 0.0 BL s^−1^ and maximum metabolic rate (MMR) estimated by extrapolating the fit to the critical swimming speed (dashed lines). (**c**) ṀO_2_ of the fish over time in a static respirometer (open circles), where the solid line indicates SMR and the dashed line indicate MMR. (**d**) Histogram of the ṀO_2_ during the static respirometry trial (black columns; bins = 2 mgO_2_ kg^−1^ h^−1^). A double Gaussian distribution was fitted to the ṀO_2_ histogram (red line), sorting SMR determined into the distribution with the lowest mean value, and spontaneous activity into the other.

A representative example of the swimming and static respirometry trials is shown in Figure 2. SMR of both LSTP and HSTP was unaffected by respirometry method and salinity treatment order (Table 1). However, there was a significant effect of time on SMR in LSTP, where SMR was 3% lower on day one than on day two (LMM, *F*_1,21.023_ = 4.444, *p* = 0.047) as well as in HSTP, where SMR was 15% lower on day two than on day one (LMM, *F*_1,20.995_ = 25.298, *p* < 0.001). Furthermore, there was a significant effect of salinity on SMR in HSTP, which was 6% lower at a salinity of 0 than at a salinity of 10 (LMM, *F*_1,20.995_ = 4.369, *p* = 0.050). MMR and AS were not affected by respirometry method, salinity treatment, time, nor salinity treatment order in either of the two populations.

**Figure 3:**
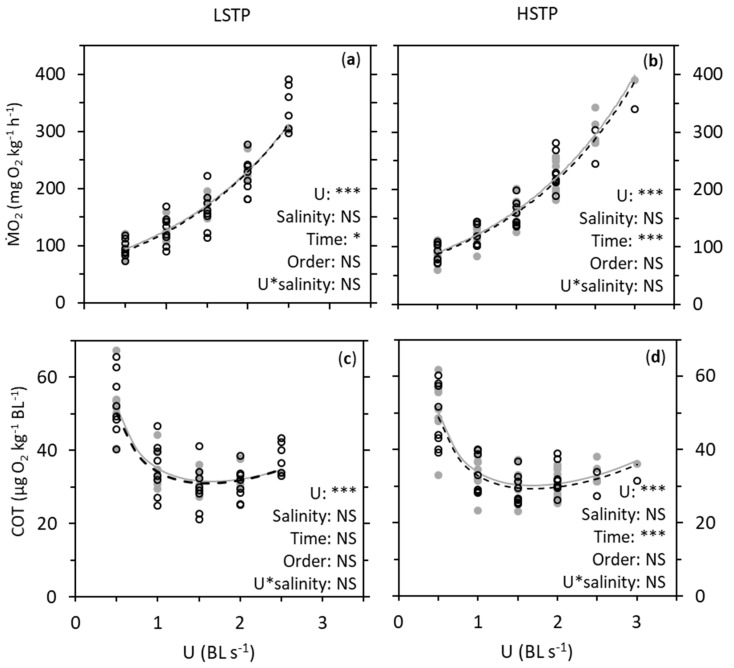
Oxygen consumption rate (ṀO_2_; (**a**,**b**)) and cost of transport (COT; (**c**,**d**)) at different swimming speeds (U) in European perch (*Perca fluviatilis*) from a low salinity tolerance population (LSTP; (**a**,**c**)) and a high salinity tolerance population (HSTP; (**b**,**d**)). “BL” abbreviates body lengths. ṀO_2_ was determined at increasing swimming speed at salinities of 0 (open circles, dashed black lines) and 10 (grey dots, grey lines). The total sample size was 16, with eight individuals being used repeatedly at both salinities. Half the individuals experienced the salinity treatments in the order 0–10 and the other half in the order 10–0. The lines in (**a**,**b**) are fitted two-parameter exponential functions of ṀO_2_ in relation to swimming speed, while the lines in in (**c**,**d**) are the two-parameter exponential functions over U. The results were analyzed with linear mixed models with respect to salinity, time (first salinity treatment vs. second salinity treatment, one month apart), the order in which the experiments were repeated on the fish, and the interaction between swimming speed and salinity. “*” and “NS” represent *p* < 0.05 and not significant, respectively.

In both populations, ṀO_2_ significantly increased exponentially with swimming speed by a coefficient of 0.613 per BL in LSTP (LMM, *F*_1,58.321_ = 400.660, *p* < 0.001) and by a coefficient of 0.596 per BL (LMM, *F*_1,61.367_ = 1020.999, *p* < 0.001) (Figure 3). There were no effects of salinity, and no interaction between salinity and swimming speed, nor salinity treatment order, in any of the populations. However, time significantly affected ṀO_2_ in both populations, being 5% lower on day two in LSTP (LMM, *F*_1,58.163_ = 5.462, *p* = 0.023) and 6% lower on day two HSTP (LMM, *F*_1,61.030_ = 139.377, *p* < 0.001). COT was significantly affected by swimming speed in LSTP (LMM, *F*_1,58.668_ = 13.342, *p* < 0.001), yet not by salinity, time, nor salinity treatment order, and there was no interaction between swimming speed and salinity. In HSTP, COT was also significantly affected by swimming speed (LMM, *F*_1,62.899_ = 21.306, *p* < 0.001), but not by salinity nor salinity treatment order, and there was no interaction between swimming speed and salinity. However, time significantly affected COT in HSTP, which was 19% lower on average on day two (LMM, *F*_1,61.153_ = 17.805, *p* < 0.001).

The power analysis showed a minimum detectable cost of osmoregulation in the present study of 10% of SMR with swimming respirometry and 12% of SMR with static respirometry (Table 2). The minimum estimated sample size to detect a significant cost of osmoregulation of 5% was 62 with swimming respirometry and 212 with static respirometry (Table 3).

Data are publicly available through the Gulf of Mexico Research Initiative Information & Data Cooperative (GRIIDC) at https://data.gulfresearchinitiative.org (doi:10.7266/N7ZW1JJ7) under the data set name “Perch swimming and salinity”.

## 4. Discussion

In contrast to our expectations, U_crit_ was not higher in iso-osmotic conditions in European perch, which were otherwise assumed favorable for the fish due to a minimal osmotic gradient between the ambient environment and the internal milieu. On the contrary, we found that the LSTP reduced swimming performance at a salinity of 10. The decrease in U_crit_ was apparently not associated with any of the measured energetic components: salinity stress did not impose a metabolic load on either SMR or ṀO_2_ during swimming, which would otherwise have implied an energetic cost of coping with salinity change and thus reduced the energy available for swimming, that is, the aerobic scope [41], in turn restricting U_crit_. MMR was also not affected by salinity change, indicating a reduction in oxygen uptake to minimize osmotic water exchange and ion diffusion over the gill epithelium (an osmo-respiratory compromise) [39], which would also have limited AS and hence U_crit_. However, the blood plasma osmolality of LSTP is known to increase between salinities of 0 and 10, while the blood plasma osmolality of HSTP remains unaffected by salinity in this range [20]. A change in internal osmotic pressure with changing salinity in LSTP could challenge regulation of muscle ion content and enzyme efficiency [63], in turn affecting muscle functionality, leading the LSTP fish to fatigue earlier at elevated salinities.

The energetic cost the cost of osmoregulation in teleosts has intrigued fish physiologists and ecologists for around half a century [31,32,34,35,37,38,54,61,64,65]. Near iso-osmotic water conditions are theoretically an energetic advantage for fish due to the added energetic cost of osmoregulation at salinities departing from this level, which could potentially explain high in situ growth rates of fish in estuaries, provide explanations for seasonal anadromous and catadromous migrations, and optimize growth of fish in aquaculture. However, in the present study, SMR and ṀO_2_ during swimming of European perch was not significantly higher in fresh water than in iso-osmotic water. We also found no evidence for the cost of osmoregulation to increase with increasing swimming speed, which we otherwise expected due to a higher potential for osmotic diffusion caused by increase in ventilation rate [31,34,35,37]. These results are consistent with a multitude of other studies which also did not find an effect of salinity on whole-animal ṀO_2_ (reviewed in [38,50]), and suggests that the cost of osmoregulation is probably modest in teleost fish.

While there is undoubtedly an energetic cost of osmoregulation, inter-individual variation may have masked the actual cost of osmoregulation. In the present study, we followed the current state-of-the-art recommendations for aquatic respirometry to increase precision and reduce variation in ṀO_2_ due to measurement noise (using intermittent-flow respirometry, fiber-optic oxygen sensors, proper respirometer-to-fish-ratios, etc. [53,66]). We furthermore employed repeated measurements on the same individuals across the experimental salinities to reduce impacts from between-individual variation. A power analysis showed that the lowest detectable level of cost of osmoregulation in the present study was 10% of SMR, and the actual cost of osmoregulation in European perch is presumably lower than this. The estimated sample size needed to circumvent the issue of inter-individual variation and potentially detect the cost of osmoregulation, which is theoretically around 5% of SMR [59,60,61], was extensive. In agreement with our analyses, a study on Atlantic salmon (*Salmo salar*) employing a sample size of 120 could not detect any difference in SMR between the iso-osmotic water and fresh or sea water [32].

Another potential concern with estimating the cost of osmoregulation by means of whole animal respirometry is that SMR of fish may not be repeatable over a longer time scale [67]. This constitutes an issue as proper salinity acclimation may take several weeks [68,69,70]. Time strongly influenced SMR and ṀO_2_ during swimming in the present study, and the issue with repeatability for measuring these metabolic rates may further mask measurements of the actual cost of osmoregulation. Interestingly, the salinity treatment order did not influence ṀO_2_, indicating no carry-over effect between the salinity treatments.

Contrary to our hypothesis, we found a slightly higher SMR at a salinity of 10 in HSTP, which indicates that the notion that the cost of osmoregulation should be lowest at near iso-osmotic conditions may not be true in all instances, and that ṀO_2_ may be influenced by other factors changing with salinity than ion regulation alone. For instance, a rise in cortisol level, at a level corresponding to the one associated with salinity change, induced increased ṀO_2_ in cutthroat trout (*Oncorhynchus clarki clarki*) parr [71]. Food intake and food conversion ration may also be affected by salinity [72], and as metabolic rates are influenced by growth trajectory [73], significant differences in ṀO_2_ between salinities may, in part, be caused by the nutritional condition of the fish. Although whole animal respirometry has been a widely applied to assess the cost of osmoregulation in fish [38,50], the many factors that potentially generate variance in ṀO_2_ determinations when measuring ṀO_2_ in relation to salinity change, combined with the presumably minor effect of salinity on ṀO_2_, render the methodology inappropriate for assessing the cost of osmoregulation compared to other approaches [59,60,61].

Earlier studies have shown that estimates of ṀO_2_ may differ depending on whether they have been measured by means of static or swimming respirometry [74,75,76,77]. In monocle bream (*Scolopsis bilineata*), for instance, SMR determined by extrapolating ṀO_2_ in relation to swimming speed to a swimming speed of 0 was different from SMR determined in a static respirometer [74]. In Atlantic cod, MMR determined with swimming respirometry during a U_crit_ trial was found to be lower than MMR with static respirometry after an exhaustion protocol (after the U_crit_ trial and manually chased) [75,76]. However, the opposite has been shown in the case of Atlantic salmon, where MMR was markedly higher when measured by means of swimming respirometry during a U_crit_ trial than when MMR was measured with static respirometry after an exhaustion protocol [77]. Such differences arising from the respirometry method may yield different results across treatments in comparative studies. The respirometry method therefore needs to be taken into careful account when planning and conducting studies to produce reliable results. Metabolic rates have been extensively used to study the effect of perturbations of fish in the wild, such as fisheries, species invasion, climate change, and pollution [78,79,80,81,82,83], and proven useful for predicting future distribution ranges of fish species and thereby aiding conservation [84,85,86]. In the present study, we found no differences in SMR and MMR of European perch when using swimming or static respirometry, and the species is thus a robust and ideal model organism for future studies on the response in metabolic rates of fish, for instance, in relation to climate change [38,54,87,88].

Our results showed intra-specific differences in the effect of salinity on swimming performance in European perch. This was also found in killifish (*Fundulus heteroclitus*) originating from populations with differences in osmoregulating capabilities, where U_crit_, similarly to our results, was lowest in iso-osmotic water in a population originating from fresh water and unaffected by salinity change in a population originating from brackish water [5]. Swimming is ecologically important for fish as it is used for migration, foraging, and escaping predators in the wild [25,26]. Enhanced swimming performance with changing salinity of sub-populations may therefore play a significant role for their ecological success in estuaries, and enable them to effectively benefit from high availability of nutritional resources and lower inter-specific competition [1,2,4].

The intra-specific differences in the effect of salinity on swimming performance found in the present study must have been caused by either a phenotypic response or a local genetic adaptation [7,10]. While we did not investigate the genetics of the fish used in the present study, earlier studies have shown differentiation among freshwater and estuarine living European perch in the Baltic Sea [17,89,90]. This indicates a genetic component behind the intra-specific differences in the physiological performance of European perch in estuarine environments, which could be an important subject for future research.

Sub-populations with enhanced ecological performance in diverging environments are potentially vulnerable due to the possibility for genetic isolation from, and loss of phenotypic plasticity in, nearby non-estuarine populations, and thereby a limited re-colonization potential from these populations [8,9]. European perch is a target for a substantial fishery in the Baltic Sea [12,13,46], where their abundance has recently declined substantially [15,18,19]. Our findings of intra-specific differences in physiological performance in relation to salinity change therefore raises conservation concerns about the species in the area. Should extinction of local populations of European perch in the Baltic Sea occur, the results of the present study also show the importance of reintroducing fish from another estuarine population if re-establishment is to be successful [91]. This may not only apply to European perch, but also to Northern pike (*Esox lucius*), which is another ecologically and economically important freshwater fish species of the Baltic Sea that is currently declining in numbers in the area [15,18,19]. The present study is therefore important knowledge for ecologists, conservation biologists, and estuarine management.

## Figures and Tables

**Table 1:** Swimming performance and metabolic rates in relation to salinity of European perch (*P. fluviatilis*) from a low salinity tolerance population (LSTP) and a from high salinity tolerance population (HSTP). The total sample size was 16, with eight individuals being used repeatedly at both salinities. Critical and optimal swimming speeds (U_crit_ and U_opt_, respectively), where swimming speed is given in body lengths (BL) s^−1^, standard metabolic rate (SMR), maximum metabolic rate (MMR), and aerobic scope are ´given by the overall means across the different response factors for each population ± SE. The statistics are from general linear models (U_crit_ and U_opt_), and linear mixed models (SMR, MMR, and AS). “*”, “***”, and “NS” represent *p* < 0.05, *p* < 0.001, and not significant, respectively.

Metric	Factor	LSTP	HSTP
U_crit_ (BL s^−1^)		2.30 ± 0.08	2.40 ± 0.09
	Salinity ^1^	*	NS
	Time ^2^	NS	NS
	Order ^3^	NS	NS
U_opt_ (µg O_2_ kg^−1^ BL^−1^)		1.68 ± 0.09	1.68 ± 0.05
	Salinity	NS	NS
	Time	NS	NS
	Order	NS	NS
SMR (mg O_2_ kg^−1^ h^−1^)		70.1 ± 2.5	69.4 ± 2.6
	Respirometry method ^4^	NS	NS
	Salinity	NS	*
	Time	*	***
	Order	NS	NS
MMR (mg O_2_ kg^−1^ h^−1^)		294.8 ± 10.7	291.8 ± 9.9
	Respirometry method	NS	NS
	Salinity	NS	NS
	Time	NS	NS
	Order	NS	NS
Aerobic scope(mg O_2_ kg^−1^ h^−1^)		294.8 ± 10.7	222.4 ± 9.7
	Respirometry method	NS	NS
	Salinity	NS	NS
	Time	NS	NS
	Order	NS	NS

^1^ The experiments were conducted at salinities of 0 and 10. ^2^ First salinity treatment vs. second salinity treatment, one month apart. ^3^ Half the individuals experienced the salinity treatments in the order 0–10, the other half in the order 10–0. ^4^ SMR, MMR, and aerobic scope were determined both by means of swimming respirometry and static respirometry.

**Table 2:** Analyses of the detectable level of cost of osmoregulation of the present study, defined as an added metabolic cost at a salinity of 0 relative to at a salinity of 10. The statistical power cut-off value was set to 0.8. The analyses were done for both swimming and static respirometry determinations of standard metabolic rate (SMR) on European perch (*Perca fluviatilis*) from a low salinity tolerance population (LSTP) and a high salinity tolerance population (HSTP).

Population	Respirometry Method	Standard Deviation (%)	Sample Size	Cost of Osmoregulation (in % of SMR)
LSTP	Swimming	8	16	12
	Static	20	16	28
HSTP	Swimming	7	16	10
	Static	13	8	19

**Table 3:** Analyses of the estimated sample size needed to detect a cost of osmoregulation of 5% SMR in the current study. The statistical power cut-off value was set to 0.8. The analyses were done for both swimming and static respirometry determinations of standard metabolic rate (SMR) on European perch (*Perca fluviatilis*) from a low salinity tolerance population (LSTP) and a high salinity tolerance population (HSTP).

Population	Respirometry Method	Standard Deviation (%)	Sample Size
LSTP	Swimming	8	82
	Static	20	502
HSTP	Swimming	7	62
	Static	13	212
